# Crystallographic snapshots of a B_12_-dependent radical SAM methyltransferase

**DOI:** 10.1038/s41586-021-04355-9

**Published:** 2022-02-02

**Authors:** Cameron D. Fyfe, Noelia Bernardo-García, Laura Fradale, Stéphane Grimaldi, Alain Guillot, Clémence Brewee, Leonard M. G. Chavas, Pierre Legrand, Alhosna Benjdia, Olivier Berteau

**Affiliations:** 1 Université Paris-Saclay, INRAE, AgroParisTech, Micalis Institute, ChemSyBio, Jouy-en-Josas, France; 2 Aix Marseille Univ, CNRS, BIP UMR7281, Marseille, France; 3 Synchrotron SOLEIL, HelioBio group, L’Orme des Merisiers, Gif sur-Yvette, France; 4 Nagoya University, Nagoya, Japan

**Keywords:** Biocatalysis, Biosynthesis, Enzyme mechanisms

## Abstract

By catalysing the microbial formation of methane, methyl-coenzyme M reductase has a central role in the global levels of this greenhouse gas^1,2^. The activity of methyl-coenzyme M reductase is profoundly affected by several unique post-translational modifications^3–6^, such as  a unique *C*-methylation reaction catalysed by methanogenesis marker protein 10 (Mmp10), a radical *S-*adenosyl-l-methionine (SAM) enzyme^7,8^. Here we report the spectroscopic investigation and atomic resolution structure of Mmp10 from *Methanosarcina acetivorans*, a unique B_12_ (cobalamin)-dependent radical SAM enzyme^9^. The structure of Mmp10 reveals a unique enzyme architecture with four metallic centres and critical structural features involved in the control of catalysis. In addition, the structure of the enzyme–substrate complex offers a glimpse into a B_12_-dependent radical SAM enzyme in a precatalytic state. By combining electron paramagnetic resonance spectroscopy, structural biology and biochemistry, our study illuminates the mechanism by which the emerging superfamily of B_12_-dependent radical SAM enzymes catalyse chemically challenging alkylation reactions and identifies distinctive active site rearrangements to provide a structural rationale for the dual use of the SAM cofactor for radical and nucleophilic chemistry.

## Abstract (web summary)

Structural and spectroscopic studies show how a B_12_-dependent radical SAM enzyme catalyses unique and challenging alkylation chemistry, including protein post-translational modification required for methane biosynthesis.

## Main

**Fig. 1:**
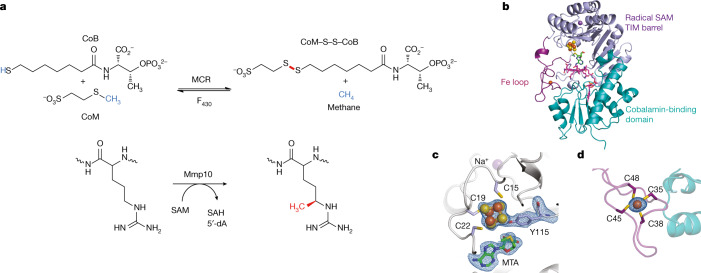
MCR and Mmp10 activity with overall structure of Mmp10. **a**, The activity of MCR producing CoB–CoM heterodisulfide and methane is enhanced by the post-translational modification of R285 catalysed by the B_12_-dependent radical SAM enzyme Mmp10. **b**, Overall structure of Mmp10 with bound sodium, [4Fe–4S] cluster, MTA, MeCbl and single iron atom cofactors (Protein Data Bank (PDB) accession 7QBT). Although Mmp10 was crystallized with SAM, only electron density for MTA was observed (Extended Data Fig. 1, Extended Data Table 1). **c**, Magnified view of the [4Fe–4S] cluster coordinated by three cysteine residues and Y115 alongside the modelled MTA molecule not coordinated to the cluster (Extended Data Fig. 1b). **d**, Iron loop with a single iron atom coordinated by four cysteine residues (Extended Data Fig. 1c). Light blue, radical SAM domain; teal, cobalamin-binding domain; purple, iron loop; green, MTA; magenta, MeCbl. The [4Fe–4S] cluster is shown as yellow and orange spheres; the single iron is presented as an orange sphere; and the sodium atom is shown as a violet sphere. Omit maps (blue mesh) of the [4Fe–4S] cluster, its coordinated Y115 and the uncoordinated MTA (**c**) or single iron atom (**d**) contoured at 3*σ* are depicted.

Methane production by anaerobic methane-oxidizing archaea is responsible for two-thirds of global methane emissions^1^, a large part of which originates from marine sediments^1^ and the mammalian microbiome^2,10^. In this process, methyl-coenzyme M reductase (MCR) has a central role by catalysing the reversible interconversion of 2-methylmercaptoethanesulfonate (CoM) and 7-thioheptanoylthreoninephosphate (CoB) to a CoB–CoM heterodisulfide and methane (Fig. 1a). The structure of MCR^3^ has revealed several distinct features for this 300-kDa (αβγ)_2_ protein complex, such as a unique F_430_ cofactor^11^ and unusual post-translational modifications^5^, including 5-*C*-(*S*)-methylarginine^4,5^, which tunes the reactivity of its active site^6,12^. Mmp10, which has been shown to catalyse this key post-translational modification^7,8^, belongs to an emerging superfamily of B_12_-dependent radical SAM enzymes^13–21^ that encompasses more than 200,000 proteins (http://radicalsam.org/)^22^. These enzymes are involved in the biosynthesis of myriad natural products including bacteriochlorophyll and antibiotics^9,16,18,23^ and catalyse  various reactions such as methyl transfer to *sp*^2^- and *sp*^3^-hybridized carbon atoms^13,14,18,24^, *P*-methylation^25^, ring contraction and cyclization reactions^26,27^. However, despite the numerous biochemical and spectroscopic studies available in the literature^13–21^, knowledge of these biological catalysts remains limited. Notably, only two structures of B_12_-dependent radical SAM enzymes have been solved thus far; however, these studies present some limitations precluding a deep understanding of their catalysis^24,27^. In addition, no structure of a B_12_-dependent radical SAM enzyme catalysing methyl transfer to an *sp*^3^-hybridized carbon atom has been reported so far, even though these enzymes are the only known biological catalysts capable of such transformation and this reaction is by far the most widespread in this enzyme family. Interestingly, these latter enzymes also have the remarkable property of being able to make dual use of SAM to initiate radical chemistry and to catalyse nucleophilic displacement, which remains poorly understood.

## Mmp10 has a unique architecture

**Extended Data Fig. 1:**
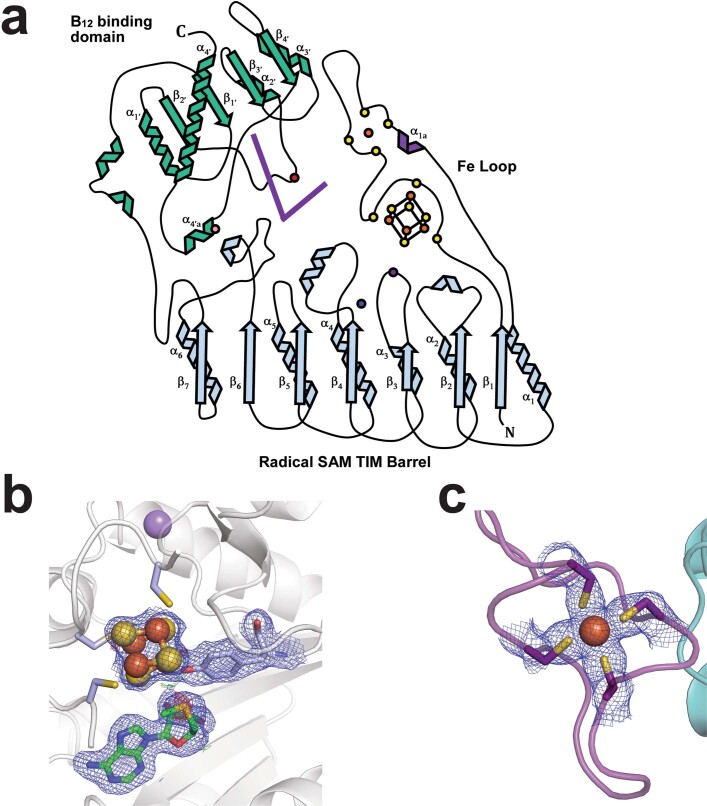
Topology diagram of Mmp10 depicting domains (a) and 2Fo-Fc and Fo-Fc maps of (b) SAM modeled as MTA, [4Fe-4S] cluster, Tyrosine 115 and (c) the single Fe ion in Mmp10 structure (Mmp10 MTA_1 structure). Three yellow circles surrounding the [4Fe-4S] cluster represent cysteine residues that coordinate the radical SAM cluster. Purple circle represents Tyrosine 115 that coordinates the fourth iron of the [4Fe-4S] cluster. Four yellow circles within the Fe-loop represent cysteine residues that coordinate a single iron atom shown as an orange circle. Red dot represents Leucine 322 that provides a hydrophobic pocket at the lower axial position of the cobalamin cofactor. Purple sticks represent cobalamin. Pink dot depicts Glutamate 378 involved in substrate binding. Blue dot shows the sodium ion. The 2mFo-DFc map is colored in blue and contoured at 1.0 σ. The mFo-DFc map is colored green (3.0 σ) and red (−3.0 σ).

**Extended Data Fig. 2:**
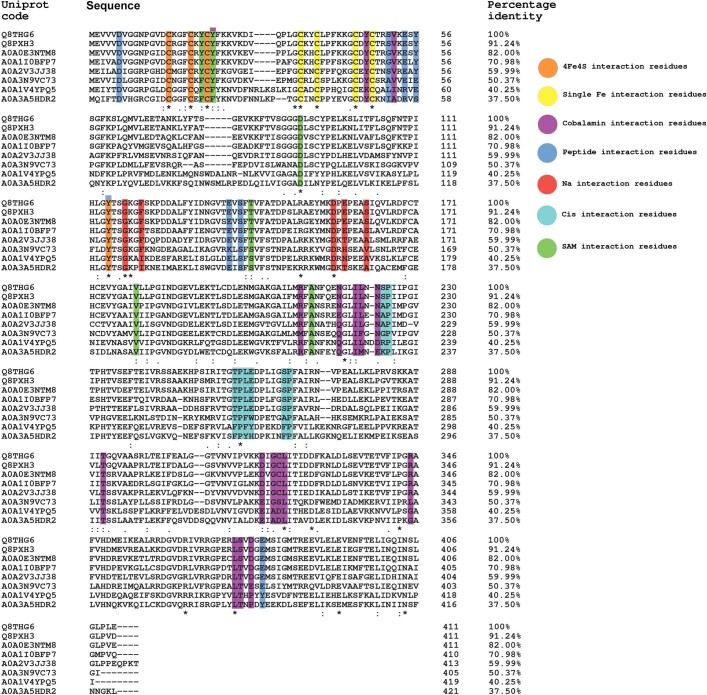
Sequence alignment of Mmp10 homologs. 258 sequences from the megacluster 1-7 (radicalsam.org), were aligned, and strictly conserved residues are highlighted in colors. For clarity, only eight representative sequences with various degree of similarity from 100 to 37% (indicated in the right column) are shown. The three cysteine residues coordinating the radical SAM cluster, the four cysteine residues coordinating the Fe-loop and Y115 are strictly conserved. Similarly, the residues involved in the B_12_-binding motif are highly conserved, notably the key L322.

The structure of holo-Mmp10 was solved at an atomic resolution of 1.9 Å, and electron density was obtained for the 411 residues of the protein (Extended Data Table 1). In a unique manner, Mmp10 is composed of two domains and an iron loop (Fig. 1b, Extended Data Fig. 1a). The first domain (residues 1–257) has an unusual radical SAM triosephosphate isomerase (TIM) barrel motif (β_7_α_6_). The radical SAM [4Fe–4S] cluster^28–31^ is coordinated by three cysteine residues (C15, C19 and C22) and, in contrast to all known radical SAM enzymes, by a strictly conserved tyrosine residue (Y115) located between the third β-strand and third α-helix of the TIM barrel (Fig. 1c, Extended Data Figs. 1b, 2). Thus far, FeS cluster coordination by a tyrosine residue has been reported for only the [FeFe]-hydrogenase maturase HydE^32^ and one nitrogenase^33^. With Y115 coordinating the radical SAM cluster, SAM is not able to bind the ‘unique iron’. However, we observed an electron density for the adenine moiety of SAM at its expected location near the top β-barrel sheet (Fig. 1b, Extended Data Fig. 1b) and coordinated by the protein backbone through hydrogen bonds. Notably, despite the atomic resolution, we could not resolve the methionine moiety of SAM, suggesting some flexibility for this cofactor, which was hence modelled as *S*-methyl-5′-thioadenosine (MTA) in this structure. This flexibility probably occurs because SAM is not coordinated to the [4Fe–4S] cluster and no polar interaction occurs with the glycine-rich motif (GGD), which usually binds the amino group of the methionine moiety of SAM^29^. Co-crystallization of Mmp10 with *S*-adenosyl-l-homocysteine (SAH) resulted in electron density consistent with the presence of the full SAH cofactor. However, among the five canonical radical SAM motifs, only a few contacts were observed, with no direct interaction between SAH and the GGD and ribose motifs. In these different structures, the adenine moiety of SAH or MTA is close to the radical SAM cluster (3.8 Å from the nearest ion of the cluster to the adenine moiety), whereas the [4Fe–4S] cluster and cobalamin (vitamin B_12_) are separated by 12 Å (from the nearest ion of the cluster to the cobalt atom).

The second unique feature of Mmp10 is the presence of a loop coordinating mononuclear iron (Fig. 1d, Extended Data Fig. 1c), which is similar to a rubredoxin iron loop^34^ although with a distinct orientation. This mononuclear iron is coordinated by a unique and conserved cysteine motif (C35, C38, C45 and C48) within the radical SAM domain (Extended Data Fig. 2). Although the presence of additional FeS clusters is common in non-B_12_-dependent radical SAM enzymes, no radical SAM enzyme has been shown to contain a mononuclear centre. Furthermore, auxiliary FeS clusters are often located in the C-terminal region and outside the TIM barrel domain, with the notable exception of BioB^35^, which is built on a complete TIM barrel.

## Roles of iron sites in Mmp10

**Extended Data Fig. 3:**
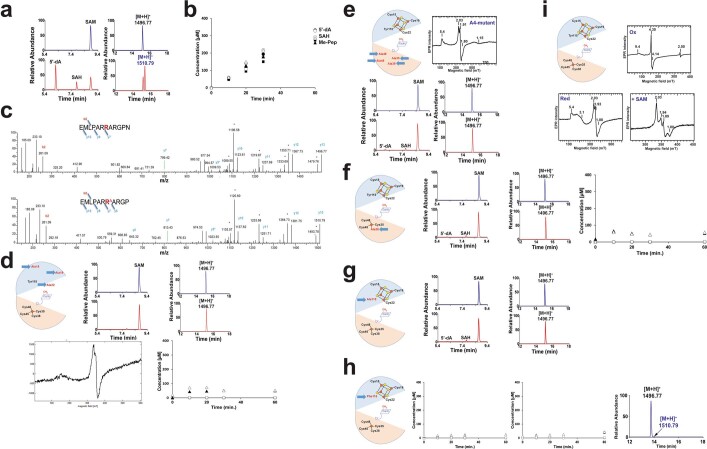
EPR analysis and activity of Mmp10 and mutants. **a**, LC-MS analysis of the reaction catalyzed by Mmp10. SAM [M]^+^= 399.14, 5′-dA [M+H]^+^= 252.11 and SAH [M+H]^+^= 385.13 are indicated. The peptide substrate [M+H]^+^= 1496.77 is converted into a methylated species [M+H] ^+^= 1510.79 (see panel c for full assignment). **b**, Time course analysis of the reaction catalyzed by Mmp10. **c**, LC-MS/MS analysis of the 13-mer peptide before (upper panel) and after (lower panel) incubation with Mmp10. See Extended Data Table 2 for full assignment. **d**, Schematic representation of the A3-mutant, biochemical characterization and EPR analysis. Right upper panel: LC-MS analysis of the reaction before (blue trace) and after (red trace) 1 hr incubation. Right lower panel: Time course analysis of the reaction. Left lower panel: CW EPR analysis. The weak signal measured at g ~ 2.0 represents adventitious FeS cluster. **e**, Schematic representation, EPR analysis and activity assay of the A4-mutant lacking C35, C38, C45 and C48 involved in the Fe-loop coordination. Upper right panel: CW EPR analysis. Lower panel: LC-MS analysis of the reaction catalyzed by the A4-mutant before (blue trace) and after (red trace) 1 hr incubation. SAM [M]^+^= 399.14, 5′-dA [M+H]^+^= 252.11 and SAH [M+H]^+^= 385.13 are indicated. **f**, Schematic representation of the C38A-mutant and biochemical characterization. Middle panels: LC-MS analysis of the reaction before (blue trace) and after (red trace) 60 min incubation. SAM [M]^+^= 399.14, 5′-dA [M+H]^+^= 252.11 and SAH [M+H]^+^= 385.13 are indicated. Right panel: Time course analysis of the reaction. **g**, Schematic representation of the Y115A-mutant and biochemical characterization. Right and middle panels: LC-MS analysis of the reaction before (blue trace) and after (red trace) 1 hr incubation. SAM [M]^+^= 399.14, 5′-dA [M+H]^+^= 252.11 and SAH [M+H]^+^= 385.13 are indicated. **h**, Schematic representation of the Y115F-mutant and biochemical characterization. Activity of the Y115F mutant in the absence (Left panel) or the presence (middle panel) of the peptide substrate. Right panel: LC-MS analysis of the reaction after 1 hr. All reactions were performed under anaerobic and reducing conditions with reconstituted enzymes and analyzed by LC-MS. Methylated peptide [M+H]^+^= 1510.79 (●,◯), 5′-dA [M+H]^+^= 252.11 (■, ☐) and SAH [M+H]^+^= 385.13 (▲, Δ) are indicated. Experiments were performed in duplicate. **i**, Schematic representation and EPR analysis of Mmp10. Ox: Oxidized enzyme as purified, Red: Reduced enzyme after anaerobic reconstitution and incubation with sodium dithionite. SAM: Reduced enzyme after incubation with SAM. g-values are indicated on the panels. In all panels, the radical SAM cluster ligated by Tyr-115, Cys-15, Cys-19 & Cys-22, the iron-loop coordinated by Cys-35, Cys-38, Cys-45 and Cys-48 and the cobalamin cofactor are indicated.

To investigate the properties of the radical SAM cluster and iron loop, we generated an A3 mutant lacking the three cysteine residues from the radical SAM cluster and an A4 mutant lacking the four cysteine residues from the iron loop, along with several mutants with individual alanine substitutions. The activity of these mutants was compared to that of wild-type protein using a synthetic peptide substrate ([M + H]^+^: 1,496.77) mimicking MCR^8^ and containing R285 (numbered as in MCR), which is the target of the methylation reaction. Mmp10 efficiently transferred a methyl group to R285 in the presence of SAM and Ti(III) citrate (Extended Data Fig. 3a–c), as shown by a mass shift of Δ*m* = +14 Da ([M + H]^+^: 1,510.79). One molecule each of 5′-deoxyadenosine (5′-dA) and SAH were produced per methylation reaction (Extended Data Fig. 3b), whereas no SAM cleavage was noted in the absence of peptide, regardless of the reductant used. As expected, the A3 mutant was inactive and unable to cleave SAM (Extended Data Fig. 3d). Mutants of the iron loop (A4 and C38A mutants) produced small amounts of 5′-dA but were unable to transfer a methyl group to the substrate (Extended Data Fig. 3e, f). Finally, when we abrogated the coordination of Y115 to the [4Fe–4S] cluster (Y115A mutant), the cleavage activity towards SAM was severely impaired and the methyltransferase activity was abolished (Extended Data Fig. 3g). Substitution of Y115 with phenylalanine only marginally restored enzyme activity (<1% of wild-type activity), which demonstrates the critical involvement of the hydroxyl group of Y115 in polar interactions following substrate binding (Extended Data Fig. 3h).

Electron paramagnetic resonance (EPR) analysis of Mmp10 revealed unique spectroscopic signatures (Extended Data Fig. 3i). First, oxidized Mmp10 exhibited a strong high-spin signal (*S*  = ^5^/_2_) at *g* = 4.30, 4.14 and 9.4, which is characteristic of a mononuclear Fe^3+^ ion, and showed a signal for [3Fe–4S]^+^ at *g* = 2.0. The latter signal corresponds to the oxidized radical SAM cluster, whereas the high-spin *S* = ^5^/_2_ signal is unusual and mirrors those reported in oxidized rubredoxin. After FeS reconstitution and reduction, signals at *g* = 2.03, 1.93 and 1.88 were noted, which correspond to the radical SAM [4Fe–4S]^+^ cluster (Extended Data Fig. 3i). In the low-field region, signals at *g* = 5.4 and 3.1 are characteristic of spin systems of *S* = ^3^/_2_ and are fully consistent with a [4Fe–4S] cluster coordinated by three cysteine residues and a non-cysteine ligation^36^. The reduced A4 mutant exhibited an EPR spectrum similar to that of the wild-type enzyme although with an altered signal of *S* = ^3^/_2_, leading to the appearance of a signal at *g* = 1.15 (Extended Data Fig. 3e). By contrast, mutation of the three cysteine residues from the radical SAM motif abrogated signal for the *S* = ^3^/_2_ species (Extended Data Fig. 3d). Finally, the addition of SAM to reduced Mmp10 led to a change in the EPR spectrum, with the development of additional signals at *g* = 1.89 and 1.80. This result is consistent with direct interaction between SAM and the [4Fe–4S] cluster^37^ (Extended Data Fig. 3i). Collectively, these data support the idea that both the *S* = ^1^/_2_ and *S* = ^3^/_2_ spin systems originate from the radical SAM [4Fe–4S] cluster and are consistent with the existence in solution of free and Y115-bound forms.

## A distinct B_12_-binding domain

**Fig. 2:**
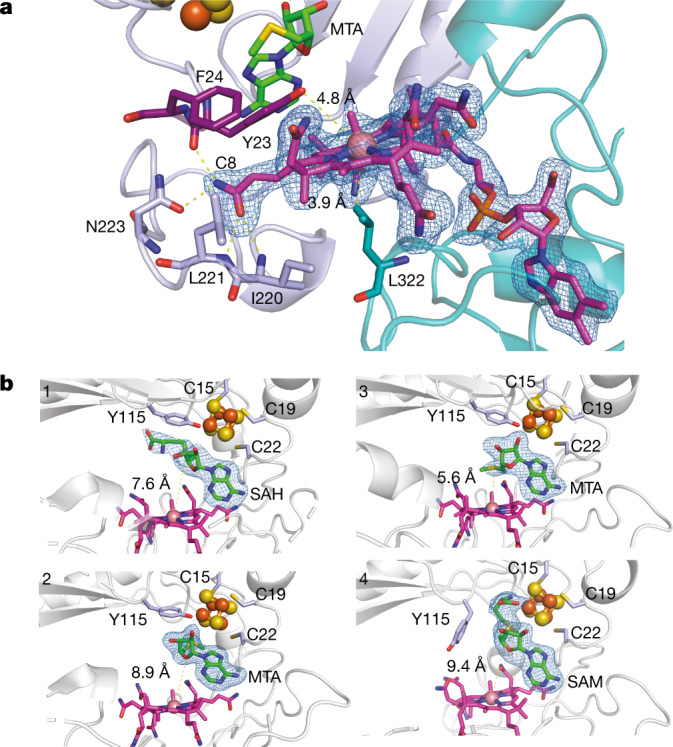
Binding of vitamin B_12_ and *S*-adenosyl ligands by Mmp10. **a**, The C8 side chain of MeCbl is shown in interaction with Y23, I220, L221 and N223 within the radical SAM domain, resulting in a planar tetrapyrrole ring. MeCbl has no lower axial ligand because it is pentacoordinated; however, L322, which resides at 3.9 Å from the cobalt atom, is part of a loop of residues forming a hydrophobic environment for the cobalt ion. Y23 appears at 4.8 Å from the cobalt ion. **b**,Snapshots of *S*-adenosyl cofactors within distinct Mmp10 structures. The distances between the sulfur atom of SAH and MTA or SAM and the cobalt ion are indicated by dashed lines. Top left, Mmp10 crystallized with SAH in the absence of peptide substrate (1: Mmp10 SAH structure; PDB 7QBV). Bottom left and top right, Mmp10 crystallized in the absence of substrate with SAM. Only the density of MTA was observed, which is labelled accordingly (2: Mmp10 MTA_1; PDB 7QBT; 3: Mmp10 MTA_2; PDB 7QBU). Bottom right, Mmp10 crystallized with SAM and its peptide substrate (4: Mmp10–SAM–peptide structure; PDB 7QBS). Light blue and purple, radical SAM domain residues; teal, cobalamin domain; green, SAM, MTA and SAH; magenta, MeCbl. The [4Fe–4S] cluster is shown as orange and yellow spheres. Omit maps (blue mesh) of ligands contoured at 3*σ* are depicted. See Extended Data Table 1 and Extended Data Fig. 4 for additional information.

**Extended Data Fig. 4:**
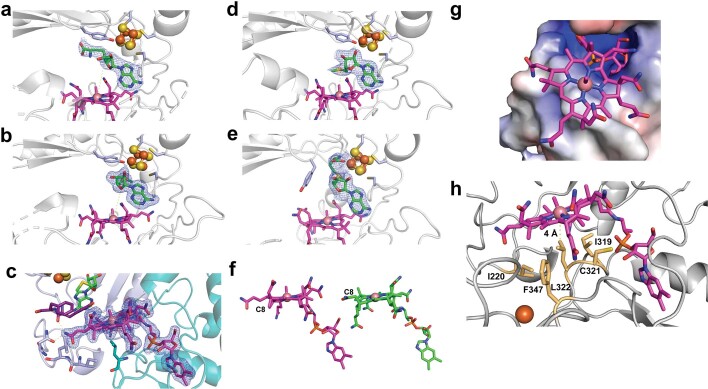
Methylcobalamin, MTA, SAH and SAM in Mmp10 structures. 2Fo-Fc and Fo-Fc maps of Mmp10 crystallized with SAH in the absence of peptide substrate (**a: Mmp10 SAH structure**). Mmp10 crystallized in the absence of substrate with SAM. Only the density of MTA was observed and labeled accordingly (**b & c**: Mmp10 MTA_1 and d: Mmp10 MTA_2 structures). Mmp10 crystallized with SAM and its peptide substrate (**e**: Mmp10 SAM peptide structure). The 2mFo-DFc map is colored blue and contoured at 1.0 σ. The mFo-DFc map is colored green (3.0 σ) and red (−3.0 σ). See Extended Data Table 1 & Fig. 2 for additional information. **f**, Cobalamin bound within Mmp10 (left; PDB 7QBT) and TsrM (right: PDB 6WTF). Cobalamin from TsrM shown in green and methylcobalamin from Mmp10 shown in magenta. The corrin ring and the sidechain C8 exhibit distinct conformations. **g & h**, Hydrophobic pocket of Mmp10 binding methylcobalamin (Mmp10 MTA_1 structure). The pocket directly beneath the cobalt of methylcobalamin has no water molecules and no charged sidechains, resulting in a hydrophobic environment. The distance between L322 and the cobalt ion is of 4 Å. Surface charge generated using APBS^66^ surface charge within PyMOL (Schrödinger).

The B_12_-binding domain (158 residues) is formed by four β-strands and seven α-helices (Figs. 1,  2). This domain comprises most of the polar bonds that hold the cobalamin dimethylbenzimidazole (DMB) tail in place. By contrast, a network of interactions from mainly the iron loop (Y23, F24 and Y47) and the penultimate loop of the radical SAM domain (R210, N217, I220, L221 and N223) coordinate the side chains of the tetrapyrrole ring. Owing to the low number of β-strands and α-helices and an absence of the polar α-helix involved in phosphate binding, this domain is only marginally related to a Rossmann fold. Furthermore, none of the canonical B_12_-binding motifs such as the His-on (DXHXXG) and SXL motifs^38^ were identified. A molecule of SAM (or SAH) and Y23 are found between the [4Fe–4S] cluster and cobalamin (Fig. 2a, Extended Data Fig. 4a–e). Y23 interacts with the tetrapyrrole C8 side chain and F24 through a π–π interaction (Fig. 2a), and its hydroxyl group is 4.8 Å from the cobalt atom (upper axial coordination), suggesting a role for Y23 in tuning cobalt reactivity. Although several charged residues have been reported to serve as a lower axial ligand in B_12_-binding enzymes either directly or through water contact, Mmp10 has a hydrophobic residue (L322) in the lower axial position of cobalamin (Fig. 2a). Because it lacks a lone pair, this residue cannot coordinate the cobalt atom. Its role is hence probably to maintain the pentacoordination of the cobalt centre and to prevent water molecules from interacting with the cobalt atom. In support of this conclusion, despite the high resolution of the structure, we observed no water molecules beneath the cobalamin cofactor, which is shielded by a hydrophobic pocket (Extended Data Fig. 4g, h). This novel binding mode is probably responsible for the atypical planar geometry of the tetrapyrrole ring (Extended Data Fig. 4f).

## Motion of SAM in the active site

No notable overall structural change was observed when Mmp10 was co-crystallized with the demethylated SAM product, with a root mean squared deviation (r.m.s.d.) of 0.37 Å over 408 residues; SAM adenine binding remained mostly unaffected. However, the distance between the sulfur atom of SAM (MTA) and the cobalt atom of cobalamin was shortened from 8.9 Å to 5.6 Å in an alternative SAM orientation (Fig. 2b, Extended Data Fig. 4). The methionine moiety is hence free to move and rotate to a distance compatible with direct methyl transfer from SAM to the cobalt atom. Following substrate binding, marked changes occurred, with displacement of Y115 coordination from the [4Fe–4S] cluster by the carboxylate and amino groups of SAM (Fig. 2b). The distance between the sulfur atom of SAM and the cobalt atom increased to 9.4 Å, whereas that between the sulfur of SAM and the [4Fe–4S] cluster shortened to 3.4 Å. These results demonstrate that SAM can adopt various conformations within the active site. Unexpectedly, coordination of the [4Fe–4S] cluster by Y115 enables the enzyme to discriminate between radical and nucleophilic uses of SAM, without requiring two SAM-binding sites.

## Peptide binding and recognition

**Fig. 3:**
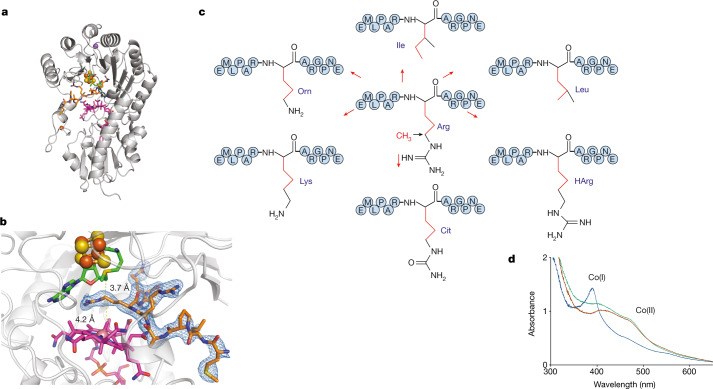
Structure of Mmp10 in complex with its peptide substrate. **a**, Overview of Mmp10 in complex with peptide substrate, shown in orange (PDB code 7QBS). **b**, Close-up of the Mmp10 active site showing SAM in green, peptide substrate in orange and cobalamin in magenta. The distance between the C5′ atom of SAM and the Cδ atom of the arginine peptide substrate R285 (3.7 Å) and that between R285 and the methyl group of MeCbl (4.1 Å) are indicated by dashed lines. The omit map (blue mesh) of peptide is contoured at 3*σ*. **c**, Sequences of the peptides used as potential substrates with the substitution of arginine (R285) with isoleucine (Ile), leucine (Leu), ornithine (Orn), lysine (Lys), citrulline (Cit) or homoarginine (HArg). **d**, UV–visible light analysis of Mmp10 pre-incubated with OHCbl. Green line, OHCbl–Mmp10; blue line, OHCbl–Mmp10 after incubation with Ti(III) citrate; red line, reduced OHCbl–Mmp10 exposed to air. See Extended Data Figs. 4–6 for additional information.

**Extended Data Fig. 5:**
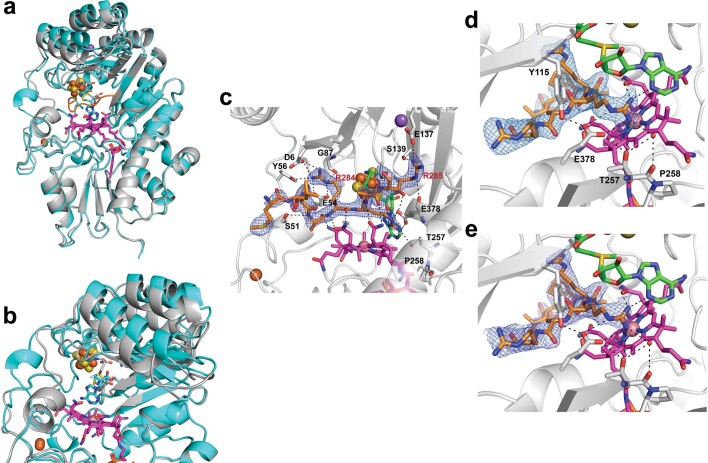
Mmp10 interaction with its substrate. **a**, Structure superimposition of substrate-free with SAH and peptide-bound with SAM structures of Mmp10. **b**, Enlargement showing the major structural movements including the α1a helix and the α1 to α4 helices of the TIM barrel. Mmp10 with peptide bound is shown in grey and without peptide in teal. Structures were aligned according to the B12 binding domain.  Alignment of the peptide-bound and substrate-free SAH structures using all domains (403 residues) has a r.m.s.d. of 0.963 Å. **c**, Polar interactions between Mmp10, its cofactors (methylcobalamin & SAM) and peptide substrate. Omit map of peptide substrate contoured at 3 σ level (in blue). SAM is shown in green, methylcobalamin is depicted in magenta, bound peptide is shown in orange, polar contacts are shown as grey dotted lines, water is shown as a red sphere. **d**, Omit map of the peptide substrate in Mmp10 active site contoured at 3 σ level (in light blue). **e**, The 2mFo-DFc map of the peptide substrate in Mmp10 active site is colored blue and contoured at 1.0 σ. The mFo-DFc map is colored green (3.0 σ) and red (−3.0 σ). SAM shown in green, methylcobalamin depicted in magenta, peptide substrate in orange.

Co-crystallization of Mmp10 with its substrate revealed clear electron density for eight residues, including R285, the target of the modification (Fig. 3a). Following substrate binding, Mmp10 adopted a closed conformation involving displacement of the α1a-helix by 11.6 Å and the α1- to α4-helices of the radical SAM TIM barrel by as much as 3.4 Å (Extended Data Fig. 5a, b). In addition to coordination of the methionine moiety of SAM to the cluster, numerous polar contacts were established involving the ribose and GGD motifs and additional interaction between Mmp10 and cobalamin. Unexpectedly, the C2, C7 and C18 side chains of cobalamin established multiple polar interactions with the peptide backbone (Extended Data Fig. 5c). At the entrance of the active site, the peptide backbone formed a sharp twist assisted by two conserved proline residues and a complex network of polar interactions between charged amino acid side chains and the enzyme backbone (D6, Y56, E54 and G87) (Extended Data Fig. 5c). R285 exhibited an extended side chain that protruded into the enzyme active site, and its Cδ atom is at the perfect distance (3.7 Å) and orientation with respect to the C5′ atom of SAM, for direct hydrogen atom abstraction (Fig. 3b). The 4.2-Å distance between the Cδ atom and the methyl group of cobalamin is also perfectly compatible with direct transfer of the methyl group from methylcobalamin (MeCbl) to the Cδ atom. Notably, the guanidinium moiety of R285 was coordinated not only by polar interaction with the protein (E378) and water contacts but also by the SAM cofactor itself through the adenine and ribose moieties (Extended Data Fig. 5d, e). Finally, Y115 became coordinated via hydrogen bonding to E378, enabling SAM to interact with the fourth iron atom of the [4Fe–4S] cluster and  radical chemistry to take place.

## Enzyme specificity

**Extended Data Fig. 6:**
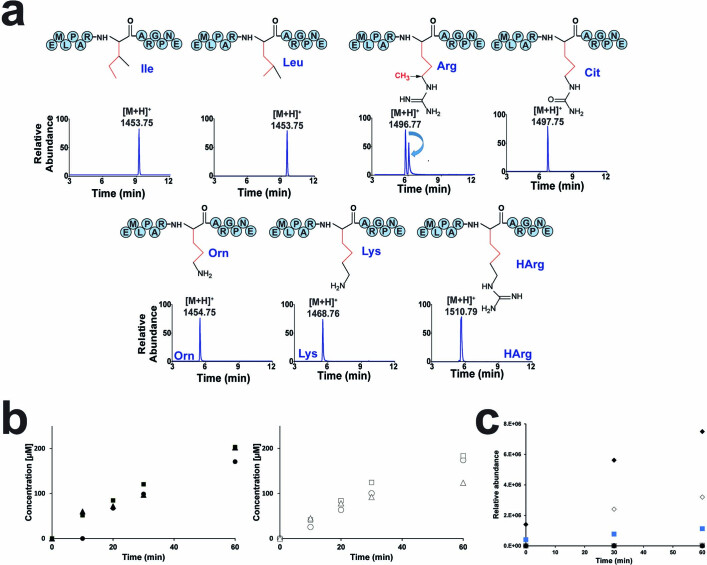
Activity of Mmp10 on peptide substrate. **a**, Influence of the critical R285 residue. Reactions were analyzed by LC-MS. Only for the wild-type peptide a methyl transfer reaction was observed (mass shift of Δm= +14 Da). See Extended Data Table 2 for peptide masses and Extended methods for reaction conditions. **b**, Activity of Mmp10 incubated with the wild-type peptide alone (left panel) or the wild-type and citrulline-containing peptides (right panel). Reactions were analyzed by LC-MS. Methylated peptide [M+H] ^+^= 1510.79 (circles), 5′-dA [M+H] ^+^= 252.11 (squares) and SAH [M+H] ^+^= 385.13 (triangles). Experiments were performed in duplicate. **c**, Formation of MeCbl by Mmp10 in the absence or the presence of Ti(III)citrate. Mmp10 was incubated under anaerobic and reducing conditions with SAM (1 mM), OHCbl (100 µM) and DTT (6 mM). Diamonds: Reaction with 1mM Ti(III)citrate. Squares: Reaction without Ti(III)citrate. Circles: Control reaction without enzyme and Ti(III)citrate. Experiments were performed in duplicate. MeCbl was detected by LC-MS analysis [M+H]^2+^: 672.80 and comparison with its retention time with authentic standard.

Mmp10 introduces only a single modification in MCR, which suggests a strict specificity contrary to that exhibited by enzymes installing multiple post-translational modifications in ribosomally synthesized and post-translationally modified proteins^9,23,39–43^. To investigate the substrate promiscuity of Mmp10, we substituted R285 with hydrophobic residues (isoleucine or leucine) or structural analogues (Fig. 3c). None of these peptides were modified by Mmp10 despite having a Cδ atom in the target side chain (Fig. 3c, Extended Data Fig. 6a,  Extended Data Table 2), including a citrulline derivative that differed from the wild-type peptide by only one atom. Competition experiments provided additional support that this analogue does not interact with Mmp10 (Extended Data Fig. 6b), which is consistent with the importance of the guanidinium moiety for interaction with E378 (Extended Data Fig. 5). In addition, E378 coordinated with Y115, which supports the idea that substrate binding acts as a switch for cluster availability. Collectively, a complex network of interactions involving water molecules and SAM, along with protein dynamics, controls the strict specificity of this enzyme. Finally, UV–visible light analysis of the hydroxycobalamin (OHCbl)–enzyme complex showed that, following Ti(III) citrate treatment, a Co(I) intermediate is formed (Fig. 3d), providing a route for MeCbl regeneration.

## Proposed mechanism for Mmp10 catalysis

**Fig. 4:**
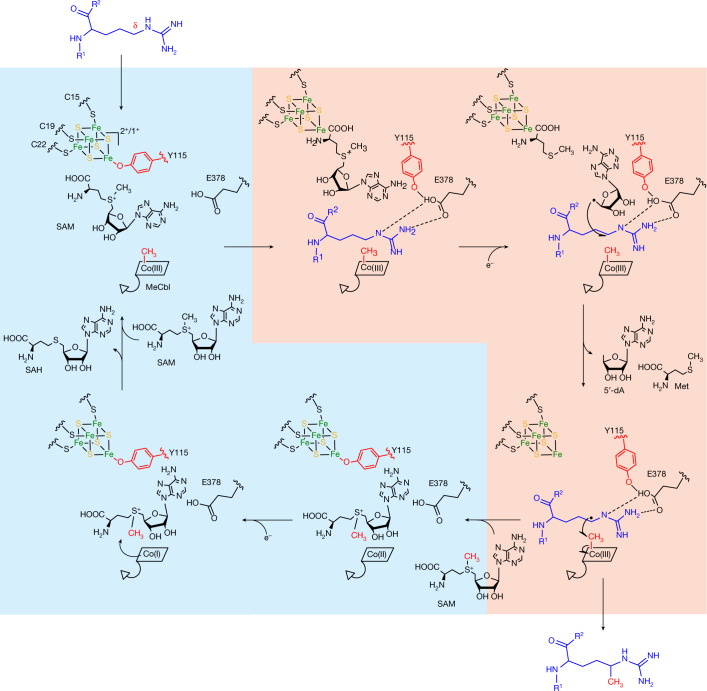
Proposed mechanism for Mmp10. Nucleophilic and radical catalysis are highlighted in blue and orange, respectively.

**Extended Data Fig. 7:**
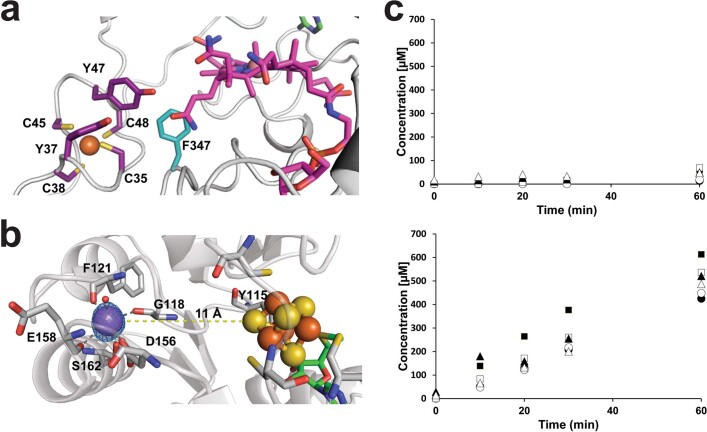
Fe loop and cation site in Mmp10. **a**, Fe loop in Mmp10 (Mmp10 MTA_1 structure). Cobalamin colored magenta, residues from Fe loop colored purple and residue from cobalamin binding domain colored teal. **b**, Cation site in Mmp10 (Mmp10 MTA_1 structure). Na ion (purple sphere) modelled in cation site with an average distance of 2.4 Å in octahedral conformation (grey dotted lines) from coordinating sidechains and water. Distance between Na ion and 4Fe4S cluster is 11 Å (yellow dotted line). Omit map of Na ion contoured at 3 σ level. 4Fe4S cluster shown as orange and yellow spheres next to SAM molecule depicted in green. **c**, Activity of the D156A mutant (upper panel) compared to the WT enzyme (lower panel). Reactions were analyzed by LC-MS. Methylated peptide [M+H] ^+^= 1510.79 (circles), 5′-dA [M+H] ^+^= 252.11 (squares) and SAH [M+H] ^+^= 385.13 (triangles). Experiments were performed in duplicate.

Interaction with the substrate, likely assisted by reduction of the [4Fe–4S] cluster, has a major role in displacement of Y115 from the radical SAM [4Fe–4S] cluster, which enables direct coordination of SAM (Fig. 4). After SAM cleavage, the formed 5′-dA radical abstracts the Cδ hydrogen atom of R285, which is at a perfect distance for direct interaction with the methyl group of MeCbl, and induces methyl transfer to R285. Then, Y115 reverts to coordination of the radical SAM [4Fe–4S] cluster, which prevents binding of a novel SAM molecule. The Co(II) intermediate generated during catalysis must be further reduced to produce the super-nucleophile Co(I) for reaction with a second molecule of SAM and to regenerate MeCbl.  Interestingly, in the absence of a strong reductant, Mmp10 can convert OHCbl into MeCbl, similar to what has been observed for TsrM^13^, albeit with lower efficiency (Extended Data Fig. 6c). Although the iron loop is ideally located and exposed to solvent, it is unlikely to have a redox potential in the range of the base-off Co(I)–Co(II) redox couple^44^, even though the potential of cobalamin can largely be influenced by the protein matrix^45^ (Extended Data Fig. 7a). At present, we favour the involvement of a ferredoxin in the reduction of Co(II).

**Extended Data Fig. 8:**
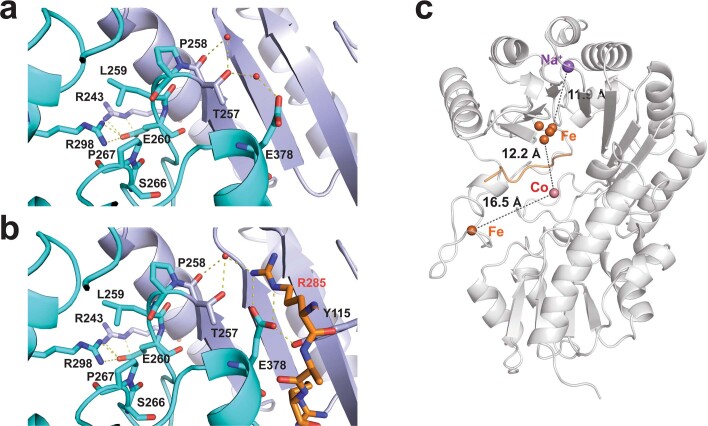
Cis-peptide bond and metallic centers in Mmp10. **a**, Cis-peptide bonds at the interface between the radical SAM and the cobalamin binding domains in the substrate free (Mmp10 MTA_1 structure) and **b**, peptide-bound Mmp10 structures. Peptide substrate shown in orange with the radical SAM domain coloured in light blue and cobalamin binding domain in teal. Polar interactions shown as yellow staggered lines. Cis-peptide bond between T257 and P258 are coordinating a water molecule which is also involved in substrate binding. The rare non-proline cis-peptide bond found between L259 and E260 is held in place through interactions between the sidechain nitrogens of R243 and R298 and the side chain oxygens of E260. **c**, Metallic centres in Mmp10. Mmp10 SAM peptide structure. Peptide substrate shown in orange.

A cation modelled as sodium is present and interacts with the residues holding Y115 in place. Its presence appears to be essential for preventing major backbone reorganization during Y115 motion, as mutation of D156, which makes key interactions in the cation-binding site, severely impairs enzyme activity (Extended Data Fig. 7b, c). The presence of a cation is reminiscent of PFL-AE^46^ and QueE^47^. However, in Mmp10, the cation is not located in the active site. Finally, four *cis* peptide bonds are present in the structure, including a rare non-proline *cis* peptide bond (Extended Data Fig. 8). These bonds are critical for the interface between the radical SAM and cobalamin-binding domains and are necessary for strict control of catalysis.

## Discussion

The structure of Mmp10, the first, to our knowledge, B_12_-dependent radical SAM enzyme catalysing protein post-translational modification, reveals the mechanism of action of these enzymes in transferring methyl groups to *sp*^3^-hybridized carbon atoms. Structural and spectroscopic analyses showed that Mmp10 contains four metallic centres at interaction distances (12–16 Å) (Extended Data Fig. 8c). We establish that Mmp10 has a C-terminal B_12_-binding domain, although residues from the whole protein are involved in binding of the B_12_ cofactor. In addition, this study reports the structure of a B_12_-dependent radical SAM enzyme in complex with its substrate properly positioned in the active site. This structure provides critical information about the structural and functional diversity of radical SAM enzymes as well as the mechanism of these complex biocatalysts that use both a radical and an S_N_2 mechanism. Major and unprecedented active site reorganization occurred following substrate binding. EPR spectroscopy and crystallographic snapshots establish that the radical SAM cluster can be transiently coordinated by a tyrosine residue, which enables the enzyme to perform either radical or nucleophilic chemistry. Recently, the structure of the B_12_-dependent methyltransferase TsrM^13,14,24^ was solved and shown to contain a [4Fe–4S] cluster coordinated by three cysteines and one glutamate residue^24^, a coordination encountered in several FeS proteins^48,49^ and proposed to preclude radical catalysis. Our study demonstrates that such coordination in Mmp10 is an intermediate state enabling dual use of the SAM cofactor.

Mmp10 contains a unique iron loop positioned beneath the B_12_ cofactor that is probably involved in shuttling electrons from the cobalt atom. In addition, a hydrophobic pocket prevents water from converting the pentacoordinated MeCbl into its more stabilized hexacoordinated counterpart, which is a strategy conserved in other B_12_-dependent radical SAM enzymes. These enzymes thus appear to have evolved unique structures and mechanisms to alkylate *sp*^2^- and *sp*^3^-hybridized carbon atoms using the twin catalytic power of the cobalamin and SAM cofactors^8,13,14,16–19,21,50,51^. In contrast to catalysis by known radical SAM enzymes, catalysis by Mmp10 requires active site reorganization and SAM flexibility within the active site. Although Mmp10 has a unique architecture among known enzymes, the role of such structural rearrangements has probably been underestimated in radical SAM enzymes, with the current work thereby delineating novel catalytic territories.

## Methods

### Protein purification

The gene for Mmp10 was commercially synthesized with codon optimization for *Escherichia coli* expression. The *Mmp10* gene was cloned into pET28-a and was transformed into *E. coli* BL21 Star (DE3) cells (Life Technologies) alongside a pRSF plasmid expressing the ISC system. Mutants were generated by gene synthesis or site-directed mutagenesis. Cells were cultured in LB with ampicillin (0.1 mg ml^−1^) and kanamycin (0.05 mg ml^−1^). Cultures were incubated at 310 K until the OD_600_ reached 0.7, at which point (NH_4_)_2_Fe(SO_4_)_2_ and 0.5 mM isopropyl-β-d-1-thiogalactopyranoside (IPTG) were added to the medium. The cultures were then cooled to 291 K and were incubated for 16 h. Mmp10 was purified by affinity chromatography in 50 mM Tris (pH 8), 400 mM NaCl and 3 mM DTT and was concentrated to ~10 mg ml^−1^.

### Reconstitution of Mmp10 and mutants

Reconstitution and all further sample preparation and experiments were performed in a glovebox in the absence of oxygen. The samples were reconstituted overnight with eightfold molar excess of (NH_4_)_2_Fe(SO_4_)_2_ and Na_2_S with 3 mM DTT for all experiments unless stated otherwise. Once reconstituted, the samples were buffer-exchanged using PD-10 columns into 50 mM Tris (pH 8), 400 mM NaCl and 1 mM DTT. For crystallography, EPR and UV–visible light analyses, Mmp10 was purified by size exclusion chromatography on a Superdex 200 Increase 10/300GL column using an AKTA Pure system. Samples reconstituted with hydroxocobalamin had tenfold molar excess added to the Mmp10 and were incubated overnight before being passed through a PD-10 column.

### Crystallization

The crystallization conditions for holo-native Mmp10 were identified anaerobically at 294 K. Initial crystals appeared after 24 h by using sitting drop diffusion and a 1:1 mixing of protein (10 mg ml^−1^ with 2 mM SAM and 200 µM MeCbl) and precipitant solutions (100 mM Tris pH 8, 20% polyethylene glycol (PEG) 8000). Holo-native Mmp10 crystals (SAH binding and alternate SAM conformation) were obtained under similar conditions. Holo-native Mmp10 peptide substrate-binding crystals were optimized using sitting drop vapour diffusion with a 1:1:1 ratio of holo-protein solution with 2 mM substrate peptide (EMLPARRARGPNE) to precipitant solution. The crystals were cryoprotected using 10% PEG 400. All were harvested anaerobically and cryocooled in liquid nitrogen.

### Crystallographic structure determination

Diffraction data were collected on the PROXIMA-1 beamline at the synchrotron SOLEIL (Saint-Aubin, France)^52^. A crystal of holo-native Mmp10 with peptide substrate (Mmp10–SAM–peptide structure) belonging to the space group *P*6_3_ was detected, and diffraction data were collected to 2.4 Å with phases obtained through multiwavelength anomalous diffraction (MAD). High-energy remote data were collected using an X-ray wavelength of 0.97857 Å and were scaled with a dataset collected at the iron absorption peak at 1.72200 Å. Diffraction images were recorded using an EIGER-X 16M detector, processed with XDS using the XDSME package^53,54^ and corrected for anisotropy using STARANISO^55^. The experimental phasing searched for one FeS cluster site, treated as a super-atom, and one cobalt site using SHARP/AutoSHARP^56^. At this stage, another unexpected separate iron site was found and was included in the phasing. Substructure determination was performed in SHELXC/D^57^ with heavy atom refinement, phasing and completion performed using SHARP^58^ and density modification using SOLOMON^59^. The model was built using several rounds of automated building with Buccaneer^60^. The final round of model building used ARP/WARP^61^, and manual building was performed within Coot^62^ with refinement by Refmac5 ^63^ and BUSTER^64^. The final model included the full-length sequence of the protein with one molecule per asymmetric unit. Subsequent data were phased by molecular replacement using PHASER^65^ with this model and with subsequent manual rebuilding and refinement as described above. A holo-native Mmp10 crystal (crystallized with SAM) with the space group *P*2_1_2_1_2 diffracted to a resolution of 1.9 Å with four molecules per asymmetric unit (Mmp10 MTA_1 structure). Another crystal (alternate MTA conformation) with the space group *P*2_1_2_1_2_1_ diffracting to a resolution of 2.3 Å with two molecules per asymmetric unit was also solved (Mmp10 MTA_2 structure). Finally, a further holo-native Mmp10 crystal (crystallized with SAH) with the space group *P*2_1_2_1_2 diffracted to a resolution of 2.7 Å and had four molecules per asymmetric unit (Mmp10 SAH structure). Data collection and refinement information can be found in Extended Data Table 1. PyMOL (version 2.0) was used in data analysis and figure generation.

### Enzymatic assay with purified enzyme

All reactions were performed anaerobically in the dark. Mmp10 reactions (100–150 µM Mmp10, 3 mM DTT, 200 µM MeCbl, 2 mM SAM, 1 mM peptide substrate, 2 mM Ti(III) citrate) were incubated at 298 K for up to 2 h and were analysed by liquid chromatography–mass spectrometry (LC–MS).

### LC–MS analysis

LC–MS analysis was performed using an ultra-high-performance liquid chromatography (UHPLC) instrument (Vanquish Flex, Thermo Scientific) connected by an HESI2 ion source to the MS instrument (Q-Exactive Focus, Thermo Scientific). Samples were diluted 50-fold in buffer A with 2 µl injected onto the column. A reverse-phase column (2.1 mm × 50 mm, 1.7 µm; Eclipse Plus C18, Agilent Technologies) was used for separation. To enhance the retention and resolution of the column, we used heptafluorobutyric acid (HBFA) as an ion-pairing agent with acetonitrile used for buffer B. All compounds eluted between 0% and 50% buffer B during 20 min at a flow rate of 0.3 ml min^−1^. Buffer A contained 0.2% HBFA in milliQ water; buffer B contained 0.2% HBFA in acetonitrile/MilliQ water at a ratio of 80/20.

### EPR spectroscopy

EPR spectra were recorded on a Bruker ElexSys-500 X-band spectrometer equipped with a standard rectangular cavity (ST4102) fitted to an Oxford Instruments liquid helium cryostat (ESR900) and temperature control system. Measurements were conducted at 6 K using a 600-mT or 800-mT field sweep range or at 15 K using a 200-mT field sweep range to focus on the *g* = 2.0 species with a field modulation amplitude of 1 mT at 100 kHz, microwave power of 10 mW and microwave frequency of ~9.48 GHz.

### Reporting summary

Further information on research design is available in the Nature Research Reporting Summary linked to this paper.

## Online content

Any methods, additional references, Nature Research reporting summaries, source data, extended data, supplementary information, acknowledgements, peer review information; details of author contributions and competing interests; and statements of data and code availability are available at 10.1038/s41586-021-04355-9.

### Supplementary information

Reporting Summary

## Figures and Tables

**Extended Data Table 1:**
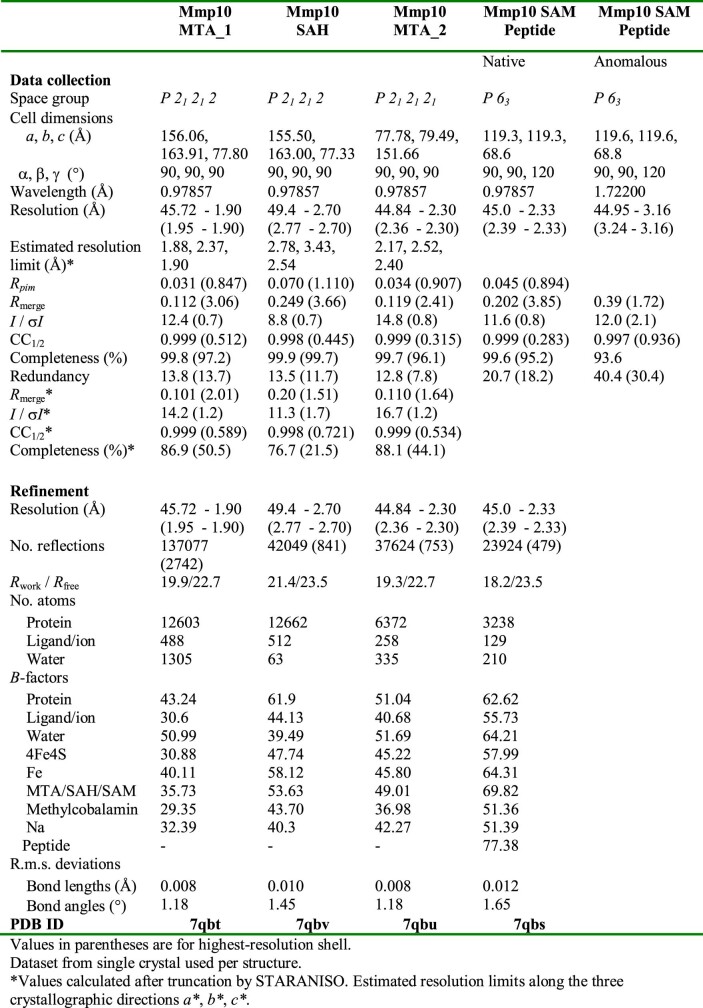
Data collection and refinement statistics

**Extended Data Table 2:**
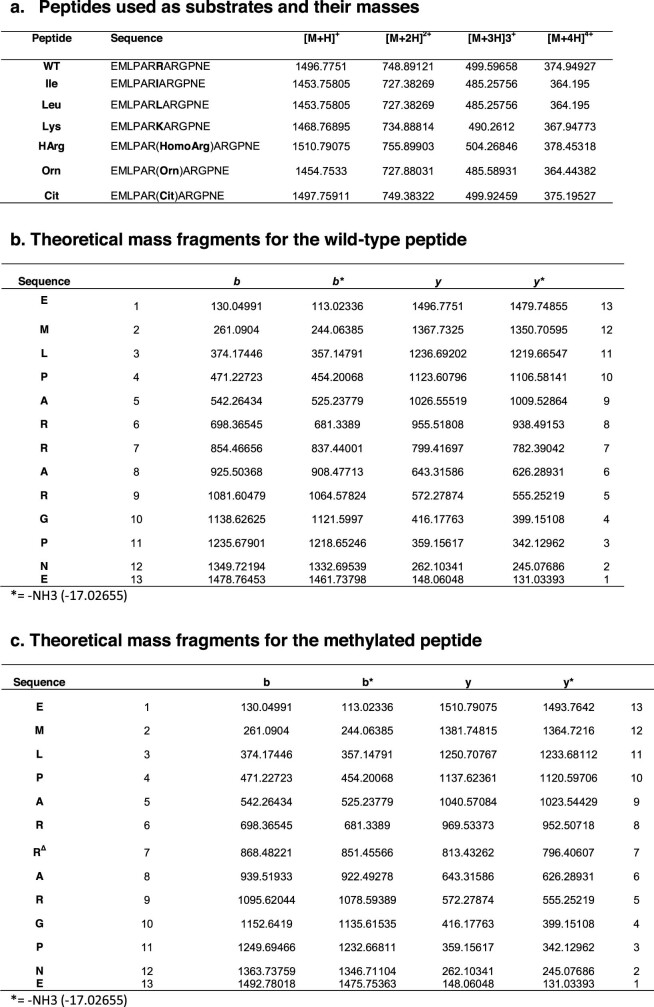
Mass spectrometry data

a. Peptides used as substrates and their masses. b. Theoretical mass fragments for the wild-type peptide c. Theoretical mass fragments for the methylated peptide.

## Data Availability

Atomic coordinates and structure factors for the crystal structures reported in this work have been deposited to the Protein Data Bank under the following accession numbers: 7QBS (Mmp10–SAM–peptide structure), 7QBT (Mmp10 MTA_1 structure), 7QBU (Mmp10 MTA_2 structure) and 7QBV (Mmp10–SAH structure).

## References

[1] Conrad R (2009). The global methane cycle: recent advances in understanding the microbial processes involved. Env. Microbiol. Rep..

[2] Tapio I, Snelling TJ, Strozzi F, Wallace RJ (2017). The ruminal microbiome associated with methane emissions from ruminant livestock. J. Anim. Sci. Biotech..

[3] Ermler U, Grabarse W, Shima S, Goubeaud M, Thauer RK (1997). Crystal structure of methyl-coenzyme M reductase: the key enzyme of biological methane formation. Science.

[4] Wagner T, Wegner CE, Kahnt J, Ermler U, Shima S (2017). Phylogenetic and structural comparisons of the three types of methyl coenzyme M reductase from Methanococcales and Methanobacteriales. J. Bacteriol..

[5] Kahnt J (2007). Post-translational modifications in the active site region of methyl-coenzyme M reductase from methanogenic and methanotrophic archaea. FEBS J..

[6] Lyu Z (2020). Posttranslational methylation of arginine in methyl coenzyme M reductase has a profound impact on both methanogenesis and growth of *Methanococcus maripaludis*. J. Bacteriol..

[7] Deobald D, Adrian L, Schone C, Rother M, Layer G (2018). Identification of a unique radical SAM methyltransferase required for the *sp*^3^-C-methylation of an arginine residue of methyl-coenzyme M reductase. Sci. Rep..

[8] Radle MI, Miller DV, Laremore TN, Booker SJ (2019). Methanogenesis marker protein 10 (Mmp10) from *Methanosarcina acetivorans* is a radical *S*-adenosylmethionine methylase that unexpectedly requires cobalamin. J. Biol. Chem..

[9] Benjdia A, Balty C, Berteau O (2017). Radical SAM enzymes in the biosynthesis of ribosomally synthesized and post-translationally modified peptides (RiPPs). Front. Chem..

[10] Benjdia A, Berteau O (2016). Sulfatases and radical SAM enzymes: emerging themes in glycosaminoglycan metabolism and the human microbiota. Biochem. Soc. Trans..

[11] Wongnate T (2016). The radical mechanism of biological methane synthesis by methyl-coenzyme M reductase. Science.

[12] Nayak DD (2020). Functional interactions between posttranslationally modified amino acids of methyl-coenzyme M reductase in *Methanosarcina acetivorans*. PLoS Biol..

[13] Pierre S (2012). Thiostrepton tryptophan methyltransferase expands the chemistry of radical SAM enzymes. Nat. Chem. Biol..

[14] Benjdia A (2015). The thiostrepton A tryptophan methyltransferase TsrM catalyses a cob(II)alamin-dependent methyl transfer reaction. Nat. Commun..

[15] Kim HJ (2013). GenK-catalyzed C-6′ methylation in the biosynthesis of gentamicin: isolation and characterization of a cobalamin-dependent radical SAM enzyme. J. Am. Chem. Soc..

[16] Wang Y, Begley TP (2020). Mechanistic studies on CysS—a vitamin B_12_-dependent radical SAM methyltransferase involved in the biosynthesis of the *tert*-butyl group of cystobactamid. J. Am. Chem. Soc..

[17] Wang Y, Schnell B, Baumann S, Muller R, Begley TP (2017). Biosynthesis of branched alkoxy groups: iterative methyl group alkylation by a cobalamin-dependent radical SAM enzyme. J. Am. Chem. Soc..

[18] Marous DR (2015). Consecutive radical *S*-adenosylmethionine methylations form the ethyl side chain in thienamycin biosynthesis. Proc. Natl Acad. Sci. USA.

[19] McLaughlin MI, Pallitsch K, Wallner G, van der Donk WA, Hammerschmidt F (2021). Overall retention of methyl sereochemistry during B_12_-dependent radical SAM methyl transfer in fosfomycin biosynthesis. Biochemistry.

[20] Zhong A, Lee YH, Liu YN, Liu HW (2021). Biosynthesis of oxetanocin-A includes a B_12_-dependent radical SAM enzyme that can catalyze both oxidative ring contraction and the demethylation of SAM. Biochemistry.

[21] Parent A (2016). The B_12_-radical SAM enzyme PoyC catalyzes valine Cβ-methylation during polytheonamide biosynthesis. J. Am. Chem. Soc..

[22] Holliday GL (2018). Atlas of the radical SAM superfamily: divergent evolution of function using a “plug and play” domain. Methods Enzymol..

[23] Benjdia A, Berteau O (2021). Radical SAM enzymes and ribosomally-synthesized and post-translationally modified peptides: a growing importance in the microbiomes. Front. Chem..

[24] Knox HL (2021). Structural basis for non-radical catalysis by TsrM, a radical SAM methylase. Nat. Chem. Biol..

[25] Werner WJ (2011). In vitro phosphinate methylation by PhpK from *Kitasatospora phosalacinea*. Biochemistry.

[26] Yang ZM, Bauer CE (1990). *Rhodobacter capsulatus* genes involved in early steps of the bacteriochlorophyll biosynthetic pathway. J. Bacteriol..

[27] Bridwell-Rabb J, Zhong A, Sun HG, Drennan CL, Liu HW (2017). A B_12_-dependent radical SAM enzyme involved in oxetanocin A biosynthesis. Nature.

[28] Berteau O, Benjdia A (2017). DNA repair by the radical SAM enzyme spore photoproduct lyase: from biochemistry to structural investigations. Photochem. Photobiol..

[29] Broderick JB, Duffus BR, Duschene KS, Shepard EM (2014). Radical *S*-adenosylmethionine enzymes. Chem. Rev..

[30] Frey PA, Hegeman AD, Ruzicka FJ (2008). The radical SAM superfamily. Crit. Rev. Biochem. Mol. Biol..

[31] Fontecave M, Atta M, Mulliez E (2004). *S*-adenosylmethionine: nothing goes to waste. Trends Biochem. Sci..

[32] Rohac R (2016). Carbon–sulfur bond-forming reaction catalysed by the radical SAM enzyme HydE. Nat. Chem..

[33] Owens CP, Katz FE, Carter CH, Oswald VF, Tezcan FA (2016). Tyrosine-coordinated P-cluster in *G. diazotrophicus* nitrogenase: evidence for the importance of O-based ligands in conformationally gated electron transfer. J. Am. Chem. Soc..

[34] Dauter Z, Wilson KS, Sieker LC, Moulis JM, Meyer J (1996). Zinc- and iron-rubredoxins from *Clostridium pasteurianum* at atomic resolution: a high-precision model of a ZnS4 coordination unit in a protein. Proc. Natl Acad. Sci. USA.

[35] Berkovitch F, Nicolet Y, Wan JT, Jarrett JT, Drennan CL (2004). Crystal structure of biotin synthase, an *S*-adenosylmethionine-dependent radical enzyme. Science.

[36] Lanciano P (2007). New method for the spin quantitation of [4Fe–4S]^+^ clusters with *S* = 3/2. Application to the FS0 center of the NarGHI nitrate reductase from *Escherichia coli*. J. Phys. Chem. B.

[37] Liu A, Graslund A (2000). Electron paramagnetic resonance evidence for a novel interconversion of [3Fe–4S]^+^ and [4Fe–4S]^+^ clusters with endogenous iron and sulfide in anaerobic ribonucleotide reductase activase in vitro. J. Biol. Chem..

[38] Drennan CL, Matthews RG, Ludwig ML (1994). Cobalamin-dependent methionine synthase: the structure of a methylcobalamin-binding fragment and implications for other B_12_-dependent enzymes. Curr. Opin. Struct. Biol..

[39] Parent A (2018). Mechanistic investigations of PoyD, a radical *S*-adenosyl-l-methionine enzyme catalyzing iterative and directional epimerizations in polytheonamide A biosynthesis. J. Am. Chem. Soc..

[40] Tang W, Jimenez-Oses G, Houk KN, van der Donk WA (2015). Substrate control in stereoselective lanthionine biosynthesis. Nat. Chem..

[41] Mahanta, N., Hudson, G. A. & Mitchell, D. A. Radical SAM enzymes involved in RiPP biosynthesis. *Biochemistry*, 10.1021/acs.biochem.7b00771 (2017). 10.1021/acs.biochem.7b00771 PMC5634935 28895719

[42] Benjdia A, Guillot A, Ruffié P, Leprince J, Berteau O (2017). Post-translational modification of ribosomally synthesized peptides by a radical SAM epimerase in *Bacillus subtilis*. Nat. Chem..

[43] Balty C (2020). Biosynthesis of the sactipeptide ruminococcin C by the human microbiome: mechanistic insights into thioether bond formation by radical SAM enzymes. J. Biol. Chem..

[44] Banerjee RV, Harder SR, Ragsdale SW, Matthews RG (1990). Mechanism of reductive activation of cobalamin-dependent methionine synthase: an electron paramagnetic resonance spectroelectrochemical study. Biochemistry.

[45] Schumacher W, Holliger C, Zehnder AJ, Hagen WR (1997). Redox chemistry of cobalamin and iron-sulfur cofactors in the tetrachloroethene reductase of *Dehalobacter restrictus*. FEBS Lett..

[46] Shisler KA (2017). Monovalent cation activation of the radical SAM enzyme pyruvate formate-lyase activating enzyme. J. Am. Chem. Soc..

[47] Dowling DP (2014). Radical SAM enzyme QueE defines a new minimal core fold and metal-dependent mechanism. Nat. Chem. Biol..

[48] Lee M (2010). Biosynthesis of isoprenoids: crystal structure of the [4Fe–4S] cluster protein IspG. J. Mol. Biol..

[49] Demmer JK (2015). Insights into flavin-based electron bifurcation via the NADH-dependent reduced ferredoxin:NADP oxidoreductase structure. J. Biol. Chem..

[50] Wang B (2018). Stereochemical and mechanistic investigation of the reaction catalyzed by Fom3 from *Streptomyces fradiae*, a cobalamin-dependent radical *S*-adenosylmethionine methylase. Biochemistry.

[51] Kim HJ, Liu YN, McCarty RM, Liu HW (2017). Reaction catalyzed by GenK, a cobalamin-dependent radical *S*-adenosyl-l-methionine methyltransferase in the biosynthetic pathway of gentamicin, proceeds with retention of configuration. J. Am. Chem. Soc..

[52] Chavas LMG (2021). PROXIMA-1 beamline for macromolecular crystallography measurements at Synchrotron SOLEIL. J. Synchrotron Radiat..

[53] Legrand, P. XDSME: XDS made easier. *GitHub*10.5281/zenodo.837885 (2017).

[54] Kabsch W (2010). Xds. Acta Crystallogr. D.

[55] Tickle, I. J. et al.* STARANISO* (Global Phasing, 2018).

[56] Vonrhein C, Blanc E, Roversi P, Bricogne G (2007). Automated structure solution with autoSHARP. Methods Mol. Biol..

[57] Schneider TR, Sheldrick GM (2002). Substructure solution with SHELXD. Acta Crystallogr. D.

[58] de La Fortelle E, Bricogne G (1997). Maximum-likelihood heavy-atom parameter refinement for multiple isomorphous replacement and multiwavelength anomalous diffraction methods. Methods Enzymol..

[59] Abrahams JP, Leslie AG (1996). Methods used in the structure determination of bovine mitochondrial F_1_ ATPase. Acta Crystallogr. D.

[60] Cowtan K (2006). The Buccaneer software for automated model building. 1. Tracing protein chains. Acta Crystallogr. D.

[61] Langer G, Cohen SX, Lamzin VS, Perrakis A (2008). Automated macromolecular model building for X-ray crystallography using ARP/wARP version 7. Nat. Protoc..

[62] Emsley P, Cowtan K (2004). Coot: model-building tools for molecular graphics. Acta Crystallogr. D.

[63] Vagin AA (2004). REFMAC5 dictionary: organization of prior chemical knowledge and guidelines for its use. Acta Crystallogr. D.

[64] Bricogne, G. et al. BUSTER version X.Y.Z. (Global Phasing, 2017) (2017).

[65] McCoy AJ (2007). Phaser crystallographic software. J. Appl. Crystallogr..

[66] Jurrus E (2018). Improvements to the APBS biomolecular solvation software suite. Protein Sci..

